# Acceptability of HIV oral self-test among truck drivers and youths: a qualitative investigation from Pune, Maharashtra

**DOI:** 10.1186/s12889-021-11963-7

**Published:** 2021-10-24

**Authors:** Amrita Rao, Sandip Patil, Pranali Pramod Kulkarni, Aheibam Sharmila Devi, Suryakant Shahu Borade, Dhammasagar D. Ujagare, Rajatashuvra Adhikary, Samiran Panda

**Affiliations:** 1 Indian Council of Medical Research- National AIDS Research Institute (ICMR-NARI), Plot No 73, ‘G’Block, MIDC, Bhosari, Pune, Maharashtra 411026 India; 2 WHO supported Acceptability project of Indian Council of Medical Research- National AIDS Research Institute (ICMR-NARI), Plot No 73, ‘G’Block, MIDC, Bhosari, Pune, Maharashtra 411026 India; 3 World Health Organization -India Country Office, R K Khanna Tennis Stadium, New Delhi, 110029 India; 4 Indian Council of Medical Research Headquarter, V. Ramalingaswami Bhawan, P.O. Box No. 4911, New Delhi, 110029 India

**Keywords:** HIV self-test, Truckers, Youths, Community voices

## Abstract

**Background:**

Ending AIDS by 2030 is a global target, to which India is a signatory. HIV-self-test (HIVST) coupled with counselling and AIDS-care, including antiretroviral therapy, has the potential to achieve this. However, national programs are at varying stages of acceptance of HIVST, as discussions around its introduction spark controversy and debates. HIV-self-test, as yet, is not part of the AIDS control program in India. Against this backdrop, we explored acceptability of an HIV oral self-test (HIVOST) among truckers and young men and women.

**Methods:**

A qualitative investigation with 41 in-depth-interviews and 15 group discussions were conducted in the district of Pune, in the western state of Maharashtra, India. These interactions were built around a prototype HIVOST kit, helped in taking the discussions forward. The software N-vivo (version 11.0) was used to manage the volumes of data generated through the aforementioned process. The study was conducted during June through December, 2019.

**Results:**

While the truckers belonged to the age bracket 21–67 year, the youths were in the age group 18–24 year. ‘Ease of doing HIVOST’ and ‘fear of needle pricks’ were the reasons behind acceptance around HIVOST by both the study groups. Truckers felt that HIVOST would encourage one to know one’s HIV status and seek help as appropriate. Accuracy of HIVOST result and disposal of the kits following use were concerns of a few. Most of the participants preferred saliva over blood as the specimen of choice. Instructions in local language reportedly would enable test-use by self. The truck drivers preferred undertaking HIVOST at the truckers-friendly ‘Khushi clinics’ or in the vehicle, while youths preferred the privacy of home. Some of the young men mis-perceived the utility of HIVOST by referring to doing a test on a partner immediately prior to sexual encounter. On the other hand, a few truckers had wrong information on HIV cure.

**Conclusions:**

Overall, the study communities expressed their acceptance towards HIV-self-test. The National AIDS Control Program, India would benefit by drawing upon the findings of the current investigation. Existing myths and misconceptions around HIV test and treatment require program attention.

**Supplementary Information:**

The online version contains supplementary material available at 10.1186/s12889-021-11963-7.

## Background

In India the HIV epidemic is on a decline. However, there are still pockets, where HIV is concentrated among the key population groups namely female sex workers (FSW; 1.56%), people who inject drugs (PWID; 6.26%), men having sex with men (MSM;2.69%) and transgender community (TG; 3.14%) [1]. This was the case even in the early days of the epidemic barring six states (Andhra Pradesh, Tamil Nadu, Maharashtra, Karnataka, Manipur and Nagaland), where a generalized epidemic was witnessed [2]. More than 1% antenatal clinic (ANC) clinic attending women, were detected with HIV in these states indicating wider spread of the virus beyond most at risk population (MARP) groups [3]. Based on such observations, the targeted intervention (TI) programs were designed, in the long run such an approach has been evaluated to be cost effective [4]. Overall goal of these TI programs in various states was to reduce the vulnerability of the above cited population groups to HIV and other sexually transmitted infections (STIs) and to establish a linkage with HIV care.

The risk of bridge populations such as long distant truckers, migrant workers and other clients of sex workers to HIV was also recognized during the early phase of the AIDS control program in India and interventions were designed to address them as well. The relevance of this approach was underlined by the epidemiologic link, which was established behind the spread of HIV from MARP groups to general population through bridge groups [5]. However, HIV intervention focus, currently directed towards bridge population is not as intense as it used to be earlier. Decreasing HIV prevalence in India in the recent past during 2005 to 2010 might be contributing to such reduced attention [6]. What is of concern though, that the HIV prevalence is refusing to go further down since 2010 [7]. Worth noting against this background is that approximately 69,000 new HIV infections took place in India in 2019 [8]. What is known is that the HIV prevalence among truck drivers is at 0.86% which is about 3 times higher than the national average adult HIV prevalence [9]. However, age specific HIV prevalence data pertaining to young adults in India is sparse. Considered together, these information not only underline that the residual risks of HIV that exist in the country, but also the need for urgent intervention development. Failing to do so, will negatively impact upon the targets of 95:95:95 to be achieved by 2030. Such targets require 95% of the people living with HIV knowing their status, of whom at least 95% will be put on anti-retroviral therapy (ART) and 95% of those on ART would achieve undetectable HIV viral load [10].

Vulnerability of truck drivers to HIV emerges from them being away from home and their regular sex partners for long period of time [11]. A study from South India in 2009 reported that truckers with higher income had higher number of sexual partners. Time away from home, urban residence, income and marital status were the factors associated with their high-risk sexual behaviour [12]. A survey from West Bengal revealed that nearly 86% of the truck drivers had sex with a casual female partner other than their wives in the last 6 months [13].

Keeping such vulnerabilities into consideration, the National AIDS Control Program (NACP) established drop-in-centers specifically at truck halt points [14]. HIV awareness program, testing, treatment facilities for sexually transmitted infections (STIs) and creating an enabling environment constituted the bouquet of services offered from these TI sites [14]. Despite such initiatives, delayed linking of truckers to HIV test and treatment has been noted [15]. As with truckers, vulnerability of adolescents and young adults to HIV in India has also been documented although they did not feature in the MARP groups. Such vulnerabilities of the youths arise either from their patronage to FSWs or exploration in unsafe drug use practices [16]. A study in 2014, among adolescents (15–19 year) in Delhi, the capital city, revealed that more than 45% of them were exposed to high-risk sexual behaviors [17]. However, studies in the recent times focusing on the vulnerability of truckers and youths to HIV have been few. Noticeably, HIV self-test has the potential to play a key role for these population groups where the threat of infection does not appear obvious to individuals.

The World Health Organization (WHO) defines HIV self-test as a process in which a person collects his or her own specimen (oral fluid or blood) and then performs a test and interprets the result, often in a private setting, either alone or with someone he or she trusts [18]. Different countries are in varying stages of acceptance of HIV self-test. Implementation of HIV self-test is still not without controversy and debate. While, several countries have already introduced or are contemplating introduction of HIV self-test as part of their national strategic plans, India, at present, is yet to do so [19]; studies focussing on acceptability of HIV self-test across different population groups have also been limited. Investigations on women in active or early labour and pregnant women, revealed that the participants preferred undertaking HIV oral self-test over blood [20, 21]. To the best of our knowledge, no studies exist on HIV self-test among truck drivers and youths in India. Against this background, we undertook this investigation to assess the acceptability of HIV oral self-test (HIVOST) among truck drivers and young adults in the country.

## Methods

The current qualitative investigation was conducted in the district of Pune, Maharashtra, during June through December 2019. Approval from the Institutional Ethics committee (IEC) of the Indian Council of Medical Research-National AIDS Research Institute (ICMR- NARI) was obtained before enrolment started. Prior to initiating in-depth-interviews (IDIs) and group discussions (GDs), written informed consent was obtained from each of the participants. INR 150 (2.5$) per person was provided at the end of each session as compensation for travel. While presenting data, we have used pseudonyms in this article to maintain confidentiality of the participants. All the enrolled participants were aged 18 year or above. An HIV oral-self test (HIVOST) kit named Morcheck, a product of Morsef Lifesciences Private Limited and manufactured by Bhat Biotech Private Limited was used as a prototype to facilitate interactions around it.

### Gaining access

Sevadham Trust is a Civil Society Organization (CSO) that works as a TI-partner of the Maharashtra State AIDS Control Society and reaches out to the truckers and migrant workers with HIV intervention. The organization has been working for this cause in Pune over the last two decades. A field assistant, with experience of working earlier with Sevadham Trust, organized IDIs and GDs with truckers from various translocation sites in Pimpri Chinchwad Municipal Corporation (PCMC).

In order to recruit young adults in the study, local youth clubs were contacted. These clubs serve as common platform where youths meet, discuss and plan activities for their future development. The field assistant contacted such youth clubs in various geographical locations to facilitate enrolment of participants with diversity. Additionally, a computer training centre, managed by a private information technology (IT) solution company, was engaged for recruitment of youths. Some of the young women were recruited through self-help groups (SHGs). Other gatekeepers engaged in recruitment of young adults in the current study were community influencers including a primary school teacher. Such recruitment approach helped us in recording different community voices.

Eligible and willing individuals were informed by the field assistants to participate in the current study. The team consisting of a moderator and a note-taker then came to the identified site for conducting IDIs and GDs. Those participating in IDIs were not invited for GDs so that wider range of responses could be obtained; GDs allowed capturing the viewpoints emerging through interaction between the group members, which otherwise would not have been possible through interviewing an individual.

### Data collection tools

The guides and probes used during in-depth- interviews and group discussions were developed by the team at ICMR-NARI based on identified domains requiring exploration and were administered in Marathi and Hindi. These tools underwent trial sessions and were modified for ease of comprehension and correctness of content (Supplementary file 1). The following domains were explored; ‘health seeking behaviour’, ‘prior HIV test experience’, ‘choice of HIV self-test’, ‘perceived advantages and disadvantages and apprehensions’. Information was also collected pertaining to the packaging of the kits, presentation, information to go with the kit, how-to-do instructions and preferred kit outlets.

### Data collection

The research team received multiple orientations on research methodology during the study period. These were conducted by the experts from social science background. Checklists prepared for IDI and GD were used by the research team (a moderator and a note-taker dedicated for a specific group of participants), which helped in ensuring consistency [22]. The field assistants ensured that the participants were from the similar socio-cultural background speaking the same native language.

Truckers were initially enrolled from Nigdi transport area, located in the PCMC area of Pune. Following completion of 8 IDIs and 2 GDs among truck drivers at this location, diverge responses ceased to be generated. The research team therefore, decided to conduct data collection at other trans-shipment locations namely, Telco Campus and Moshi. IDIs and GDs were conducted either at the transport offices or in a temple premise identified by the field assistants. A total of 16 in-depth-interviews (IDIs) and 6 groups discussions (GDs) were completed with the truck drivers.

Twenty-five IDIs (12 with young women and 13 with young men) and 9 GDs (4 with young women and 5 with young men) were conducted with the youths. Most of the IDIs and GDs took place either at rented locations or community halls or residence of the participants or project office premise as preferred by the participants. Privacy was maintained at each of these interaction sites; ensuring that no observing bystanders were present during these sessions.

### Data analysis

All interview and group discussion sessions were audio recorded and transcribed exact and translated in English. This approach of using English as a common language was to capture the essence of the responses. Data analysts subsequently checked for the quality of translations so that the nuances expressed by participants in local languages were not missed. We incorporated the Guba and Lincoln criteria to check for the quality of research (Table 1) [23]. Acceptability of HIV self-test was explored by carrying out content analysis of both the transcribed and translated responses. A separate codebook was developed for each of the study groups based on the domains explored. Short descriptions of the codes and quotes around them was compiled. This was reviewed periodically and reorganized to identify major or minor themes (categories). The data analysts, with assistance from the research supervisors and other team members helped to resolve the coding discrepancies, if any. Among the other methods, in-vivo coding was used to capture the voices and concerns of the study participants. The field notes prepared by the interviewers before and after their interactions with study participants helped in gathering sufficient reflexivity. N-vivo software (version 11.0) was used to help in organizing the responses and subsequent analysis.

**Table 1:** Strategies adopted to ensure quality in the present HIVOST acceptability study

Rigour Criteria^a^	Purpose	Original Strategies	Strategies applied in our study to achieve rigour
Credibility	To establish confidence that the results (from the perspective of the participants) are true, credible and believable.	• Field assistants chosen from the local community played a crucial role in engaging the participants for the IDIs and FGDs	• Prior experience of the field assistant in working with truckers and self-help groups helped us in conducting quality IDIs and FGDs against a backdrop of trust and rapport.
		• Interviewing process and techniques	• Research team members conversant with the local socio-cultural context used the guides and probes in mock sessions and received inputs for modifications as needed. Place of interactions between the researchers and participants were also chosen as per participants’ convenience ensuring privacy and confidentiality
		• Establishing investigators’ authority	• The research team received multiple exposures on research methodology during the study period. Orientation programs were also conducted by experts from social science background so that everyone was well versed with the methodology applied
		• Collection of referential adequacy materials	• The field assistant and interviewers filled the field notes and debriefing forms at the end of each session. This helped the entire team to conduct ongoing and final analysis, and adopt inductive investigation approach.
		• Peer debriefing	• Debriefing sessions were held with the research supervisor and colleagues; which helped in clarifying doubts and resolving conflicts around coding (if any)
Dependability	To ensure the findings of this qualitative inquiry are repeatable if the inquiry occurred within the same cohort of participants, coders and context.	• Rich description of the study methods	• Detailed draft of the implementation plan is available.
		• Establishing an audit trail	• Information obtained from earlier cohorts of participants (truckers in particular) were contrasted against the latter cohorts to examine if saturation point was being reached through repeatability and also to record rare points of views.
		• Stepwise replication of the data	• While truckers were recruited from three different halt points, responses were contrasted to examine replication. Youths recruited from different clubs or locations similarity offered opportunity for such comparisons.
Confirmability	To extend the confidence that the results would be confirmed or corroborated by other researchers.	• Reflexivity	• We implemented reflexive notes and also held weekly investigators’ meetings.
		• Triangulation	• Several triangulation techniques (IDIs vs GDs, data sources such as transcript, field notes and debriefing notes) were used in the current investigation to obtain comprehensive views around the domains explored
Transferability	To extend the degree to which the results can be generalized or transferred to other contexts or settings.	• Purposeful and convenient sampling to form a nominated sample	• We used different data collection approaches to capture voices from the communities including common and rare point of views, with potential for their transferability to similar socio-cultural settings
		• Data saturation	•In order to identify if data saturation was reached, we carried out 8 to 10 or more interactions and also had on-going analysis.

^a^Criteria proposed by Guba and Lincoln were used [23]

## Results

### Profile of the participants - truckers

While some of the truckers were engaged in long-distance travel across the country, movements of others were limited to the State of Maharashtra. All the IDI participants among truckers were married and belonged to the age group 21 to 67 year. While one of them was a science graduate, six had primary school level education, 4 had studied till 10th standard and the remaining 5 had completed 12th standard of school education. Each of the interviews lasted for 50–85 min. Each focus group with the truckers had 5 to 6 participants and most of the discussions lasted about an hour; the longest taking 1 h and 40 min. The discussants belonged to the age group 21–55 year; most were married and the level of school education varied from 3rd to 12th standard.

### Profile of the participants – youths

All the participants were in the age group of 18 to 24 year. Majority of the women interviewees had secondary school education and some completed graduation. Among the 12 young women interviewees; only 4 were married and home-makers. Among the unmarried women, most were studying, one was at home and only one of them was involved with income generating activity.

All the participating young men in IDIs and GDs were unmarried. Of the 13 young men enrolled for IDI, 3 did not have school education beyond higher secondary (12th standard) and four had completed graduation in subjects such as arts, science and engineering; five others were continuing graduate studies. In-depth interviews with women lasted for 50 min to 1 h, while interviews with men lasted for 30–50 min.

Four GDs were held with young women (3 groups constituted by unmarried women and 1 by married women); each involving 5 to 6 participants. Most of the women were studying;10th standard to pursuing a degree at college; 2 of them were involved with income generation activities. Each of these GDs lasted for about 45 min.

Each of the five GDs with young men lasted for about 65 min and each involved 4 to 7 participants. Their level of school education varied from 10th standard to graduation. Members of only one group were involved with income generating activities, while the rest were all students.

### Prior HIV test experience

Majority of the truckers had undergone HIV test; some at ‘Khushi clinics’ - an initiative by the Transport Corporation of India Foundation aiming to reduce spread of HIV and other STIs among long distant truck drivers. Others took HIV test at government hospitals. While overall experience in the former setting was positive, hurdles were faced by some in government settings. ‘Apprehension of getting identified while seeking HIV test from public health facilities’ and ‘spread of such information within the groups of truckers’ were associated concerns.

*“He is afraid of going to hospital. What he says – individuals will see him – that’s why, need not go to hospital – that’s why he does not come in front”*

*-* Igat, 45 year (TRUCKER 11)

All married women IDI-participants (during pregnancy) and 2 unmarried women had HIV test experience. A few women members in GDs had prior HIV test experiences as well. Contrastingly, among the male participants, only one interviewee and 2 group discussants reported experience of prior HIV test.

Difficulties faced by women during HIV test were of varying nature such as pain associated with needle prick during blood drawing, panic associated with the volume of blood drawn, fear of unknown as HIV test was undertaken for the first time and the hassle of detour from one department to another in the hospital. Negative attitude of some of the health care providers was another concern.

“*That you know – take blood from finger, take blood from vein – I had much trouble – take a lot of blood – they remove blood until tears come in eyes … … I had never taken HIV test before delivery – but during pregnancy they took a blood sample … but then I was scared a bit”*

- Suna, 24 year (YOUNG WOMAN 02)

### Myths and misconceptions around HIV

Interviews unravelled a range of mis-information truckers had on HIV transmission and treatment. Reportedly, a newspaper in Osmanabad (an administrative district in Marathwada region in the state of Maharashtra) published a story on an HIV infected individual getting cured following consumption of pesticides used on cotton plants. An incident of HIV cure by snake venom was narrated by another trucker. None of the youths reported any such information around HIV.

### Advantages of HIV oral self- test

Most of the truck drivers felt that HIVOST would benefit them and a few of them opined that availability of such a kit would lead to increased detection of infection and further linkages with anti-HIV treatment. Vulnerability of the truck drivers to HIV and other diseases were highlighted during these sessions, and ‘confidentiality’ as well as ‘convenience of doing the test by self’, were perceived as merits of HIV self-test.

“*The advantage is that if we have any information then we will implement … . If we have to walk on the road and we know the direction of the road then only we will reach … if we do not know the direction of the road then we will get lost … that is why I have interest in it”*

- Duey, 61 year (TRUCKER 04)

“ … .*as many drivers are there, they all will benefit … actually, means driver line is such that people have to go outside for work … all these diseases happen mostly to driver people … mainly drivers are targeted … It (HIV self-test) will be useful for us … it will be very useful for those in this field”*

- Suaj, 57 year (TRUCKER 14)

HIV oral self-test (HIVOST) concept was greeted by most of the young adults during IDIs and GDs. Long queue, fear of getting identified by somebody known, stare from people, and fear of breach of confidentiality at HIV testing centres were the reasons cited by young men welcoming HIVOST. Some of them even indicated that HIVOST would enable one to take the test at home and highlighted the ease of using it.

“*We can easily take saliva – similarly blood we cannot draw”*

- A young woman from group discussion

(GD YOUNG WOMAN 03)

*“Because we can do it easily … because saliva is in our mouth, we can easily do the test, blood is in our body … we have to draw it. And some people are scared of blood that is why saliva is comfortable”*

- Gash, 18 year (YOUNG MAN 08)

Advantage of using oral saliva-based test in children was highlighted by a married woman, while another woman elaborated upon hesitation to discuss HIV even with a doctor and highlighted the advantage of HIV self-test in such context. Women participants, without prior HIV test experience, reflected upon their social interactions and highlighted how HIVOST could be helpful. Situations cited by them encompassed young boys and girls contemplating sex, pre-marital HIV test and familial level disputes. Some of the young men mentioned that HIV self-test should be deployed in brothels and lodges before engaging in sex (wrongful expectation).

“*If – I get an easy option (choice) at home itself, then I will not waste (spend) my time by going to the lab – If facilities are provided, then definitely there is no harm in taking them – why to waste our time – while going outside, waiting in queue for reports”*

*-*Shpa, 23 year, *(*YOUNG WOMAN 04)

*“I can do the self-test and it would be confidential. I can directly discuss with the doctor - there is no need to tell anyone, or no need to write anywhere”*

-Psad, 21 year, (YOUNG MAN 07)

### Specimen of choice

Saliva was preferred over blood by both the truck drivers and youths. ‘Ease of using saliva as test specimen’, ‘lack of knowledge on how to draw blood’, ‘quick results’, ‘ability to do the test by self’ and ‘fear associated with needle prick’ were cited advantages. Young women preferred HIVOST compared with the traditional blood-based HIV test conducted in laboratories or hospitals; a few of them did not express any preference for clinical specimen, and very few preferred blood over saliva.

*“There is no need to tell anyone – we ourselves can come to know if we have HIV or not” -*Gan, 28 year (TRUCKER 07*)*

In two of the group discussions with young men, rare viewpoints such as presence of HIV in the blood and unknown accuracy of saliva-based test were put forth as justification to choose blood over saliva. The other rare justifications were blood being internal (core) element of the body would capture the presence of the disease at an early stage and possibility of using the blood specimen taken for HIV test to conduct other investigations such as CD4 count.

*“If doing from blood then you can know HIV, but you can also know CD4 count … .or other things can be checked”*

- Deti , 18 year (YOUNG WOMAN 03*)*

### Apprehensions around HIVOST

We grouped potential harms as perceived by the participants in three categories, a) societal, b) individual and c) environmental.

### Societal harm

None of the youths during IDIs or GDs flagged societal harm as an issue that could arise following introduction of HIVOST. A few though talked about judgmental attitude of the society towards HIV.

“*Means, as now what I said as the logo is very famous (HIV logo in the form of a red ribbon) because where ever HIV word comes … at that place, this ribbon comes ..., so basically this logo belongs to this (HIV). So may be someone notices this, so the person in front may quickly start to think that the person has done something. So, he needs this product. Sometime people do not think, that why are you doing this – Means when a person sees something in front of him, so he quickly starts to think about it”*

- Shpa, 23year (YOUNG WOMAN 04)

“*If we share about it with friends that I have such and such problem... even he is very close to us, he may have friends to whom he can tell ... that your HIV test result is so and so... then people start looking at you in a different way. And due to this fear, one cannot talk about it (test) or one avoids to take this test”*

- Roan, 21 year (YOUNG MAN 01)

### Individual harm

Possibility of getting into depression leading to self-harm or reluctance to seeking treatment following positive HIV result were apprehensions raised by some of the youths (both men and women).

*“That person will be mentally disturbed – he will not understand what to do. If some person is too emotional then he will do something to himself – means suicide etc. Some person will go into depression (after HIV OST positive result)”*

- Prti, 19 year (YOUNG WOMAN 09).

Anxiety related to HIV positive test result was obvious through the responses of a few truckers; one of the truckers viewed HIV positive test result as something like the end of the world. These were not specific to HIVOST though.

*“They will be demoralized as soon as they get to know … first and foremost they would get demoralized. Sometimes, they would say that they don’t even want the tablets … since now, I have got the disease … I don’t need to take the tablets … it’s better to die … if society comes to know about it tomorrow that I have got this … then who do I face … what would I tell people at home”*

-Isa, 47 year (TRUCKER 05)

Young men also brought up issues such as ‘leakage of information’ and ‘forced testing during marital negotiation’. Very few participants expressed that prank could be played with HIV self-test. Accuracy of the HIV self-test result was another concern raised by some of the young men and women

“ *… … if I want to get someone into trouble – if I want to demoralize someone I will do someone’s HIV test … one who is HIV positive and push it on you … and say you are HIV positive... then in your mind you’ll have doubts about HIV - you will be demoralized a little bit – the freshness that you had or that you were so free – you will not remain that free – in your mind there will be continuous worry”*

- Psad, 21year (YOUNG MAN 07)

“*I don’t know about its accuracy – It’s new test right. You will have to prove it first then only you can bring it in market. Everyone should be aware of its accuracy*”

- A young man from a group discussion

(GD YOUNG MAN 01)

A rare point of view was raised pertaining to the fluid (reagent) in the tube that comes with the HIVOST-kit, by a truck driver from Punjab. He mentioned that the fluid could be used for injections by drug users while, a young male interviewee assumed that the fluid might contain acid and could be poured on to someone to cause harm.

### Environmental harm

A few youths were concerned about safe disposal of the test kits, failing which could result in environmental pollution. A few truckers were also concerned about disposal of test kits vis-à-vis environmental pollution. Inappropriately disposed self-test kits in the garbage was seen by another truck driver as an issue concerning risk to others’ health.

*““Little bit it will affect the environment, and also pollution will take place – because we throw it in the garbage – basically there is not one as such – if you go to their work place and see that they set it on fire, and from that air pollution is caused,”*

*-*Shpa, 23 year *(*YOUNG WOMAN 04)

*“If this test comes in the market then everyone will know about it – we can’t throw it there. Someone will see it while emptying the garbage there or while throwing the garbage if someone sees it—they will come to know”*

*-*Isa, 47 year, (TRUCKER 05)

### Kit considerations

Participants were shown various components of the HIV self-test kit and the package insert with pictorial diagrams on ‘how-to-do’ steps. The following domains pertaining to kit consideration were explored (Table 2); a) packaging, b) instruction leaflet, c) kit outlet, d) cost consideration, and e) information to go with the kit.

**Table 2:** Kit considerations

DOMAINS	QUOTATIONS
**PACKAGING**	*“Kit looks a little big, sir ... It should be a little small ... one that can easily fit in the pocket”* *-*A truck driver from group discussion (GD- TRUCKER 04) *“Name (what for) should be written only. -it should be known only”* - Prik, 23 year (YOUNG MAN 11*)*
**HOW TO DO IT - INFORMATION**	*“I would like to know if someone explains. But if pictures are displayed in a different way then it’s okay. If it is shown in hospitals how to do it, it would be much better. If explained in local Marathi language then it would be of benefit”* - Igat, 45 year (TRUCKER 11)
	*“As you can see in “Save the girl child and educate her (Beti bachao, Beti Padhao) … .on the television … in the same way it (HIV self-test) can also happen … it should happen for this (HIVOST). That means every home...it should be known that there is such kind of thing is there. While sitting at home you can check / test. For this more advertisement should be made”* - Shpa, 23 year (YOUNG WOMAN 04)
**ACCESS POINTS**	*“It should be available everywhere – at Dhaba, at Pan Stall. It should be made available in the hospitals and even at your office – your department – It should be available in all these places”* - Diar, 43 year, (TRUCKER 12)
	*“Even at schools and colleges – as youths must be aware - It is very good for young people. They should be aware of such things”* - Prla, 23 year, (YOUNG WOMAN 11)
**COST**	*“If it is kept for free then it would not be so much – people would not understand its importance – so at least some price should be fixed”* - Nene, 28 year (TRUCKER 07)
	*“If free, the one who is in need will take it and the one who does not need it will also take it.”* *-*A young boy from group discussion (GD-YOUNG MAN 03)

A few youths expressed that the kit should have been smaller in size for convenience of carriage. Most of the participants wished to see HIV symbol (red ribbon) on the kit. It was further suggested that the instructions should be presented in local language for easy comprehension.

Most of the youths considered taking HIVOST in the privacy of their bedroom or other places home as appropriate, while truckers preferred taking it at ‘Khushi clinics’ or in the vehicle. Easy availability of the kits at various halt points such as ‘toll nakas’ and ‘dhabas’, were the felt needs of the truck drivers. Youths recommended making kits available at schools and colleges. Wastage and mischievous use of kits, when made available free of cost, were the apprehensions of the youths and truck drivers. The suggested price tag for a kit ranged from INR 10 to INR 400.

## Discussion

This qualitative investigation explored issues around HIV self-test from the perspectives of truckers and youths; two vulnerable population groups in India. The country is committed to the global goal of ‘ending AIDS by 2030’ [24] and the National AIDS Control Program (NACP) in India is working towards it. However, the major challenge is to encourage people to know their own HIV status, could be linked with anti-retroviral treatment facilities. A systematic review of 23 studies by Figueroa et al. reported high acceptability of HIV self-tests among key population groups such as MSM, FSW, TG, PWID and people in prison [25]. The issue nonetheless has not been studied adequately among certain vulnerable populations such as youths and truck drivers. The present investigation, being qualitative in nature and examining acceptability of HIVOST, therefore stands out as an important initiative.

Both truckers and youths, during our inquiry, welcomed HIV self-test. Truck drivers from Hyderabad in Andhra Pradesh accepted oral HIV self-test as an innovative HIV intervention approach. Most of the truck drivers were even willing to undertake HIV self-test while available [26]. Studies focusing on HIV self-test among youths in India are sparse. A qualitative study from Malawi and Zimbabwe among 16- to 25-year-old reveals that HIV self-test was preferred provided it was available free or at low cost [27].

In the current investigation, saliva-based HIV self-test was preferred over blood. The advantages cited were freedom from fear of needles and ability to do the test by self. A study conducted among in-patients and out-patients at the rural teaching hospital in Sevagram, Maharashtra recorded preference for oral-fluid over the blood-based HIV self-test [28]. However, scepticism on accuracy of HIVOST and the need for establishing linkages with the post-test counselling services were expressed by some of the participants in the current investigation as was noted by other researchers [29–31].

The present investigation revealed the discomfort of the participants to go to government hospitals for HIV testing; loss of privacy and apprehension about breach of confidentiality were the concerns. Contrastingly, truckers undergoing HIV test at Khushi clinics, located at trans-shipment sites, found the arrangement user-friendly. Woodford et al. elaborated upon the barriers to HIV testing at individual, inter-personal, socio-structural and health care levels among the MARP groups in Chennai, Tamil Nadu in India. Stigma associated, not only with the disease, but also HIV-test per se was cited as hindrance to access test services. Participants in this study also feared discrimination from the community and society, which delayed their access to HIV-tests. Timings, long queue, and extended waiting period were other deterrents for accessing these facilities [32], which were similar to our findings. Noticeably, most of the youths except young married women (who got tested for HIV during ante-natal check-up), in our study did not have a prior HIV test experience.

The participants in the current study reported some apprehensions around HIVOST such as accuracy of the results, disposal of kits, and self-harm. These findings were similar to the concerns raised by the MSM and TG populations around in Pune, Maharashtra in India [33].

Truckers, in the current investigation, preferred obtaining instructions on HIVOST through live demonstration or video clippings and youths wished to have them in local languages. Some of the youths erroneously felt that HIVOST could be used as a diagnostic test rather than a screening tool. On the other hand, the myths and mis-conceptions around HIV treatment were limited to only a few of the truckers. Given the practice of truckers spending time in groups at halt points, and wrong information could spread through words of mouth, interventions to dispel them appeared important. Studies published nearly a decade ago, similarly highlighted misconceptions prevailing among truck drivers, on the mode of transmission [34]. It was intriguing to note that in a study focussing on youths recorded beliefs that HIV could be cured and a vaccine was available for HIV [35].

During current inquiry, the truck drivers wished that they could get HIV self-test kits at common halt points; reportedly, they would prefer using them either at the trucker-friendly-clinics (such as Khushi) or in the vehicle. A study from Kenya, among truck drivers, recruited from wellness clinics, highlighted that those, who never undertook an HIV test would prefer HIV self-test [36]. Young adults in the current study mentioned that they would prefer taking HIV self-test in privacy of their homes. This was similar to the findings emerging from South Africa. In Mozambique, adolescents preferred taking the test at youth-friendly clinics [37–39].

HIV self-test has been in discussion for more than a decade [40]. However, a number of countries are still weighing the pros and cons of introducing HIV self-test in their policies. India is still debating its inclusion in the program. We hope that the evidence generated across different population groups, as with the current investigation, assist the national program in implementation of HIV self-test.

## Limitations

This is a small-scale acceptability study that was conducted among truckers and youths in the district of Pune, representing only a sub-section of the study population. We enrolled truckers halting at the translocation points, which were selected as recruitment sites. However, such recruitment strategy could not include the truckers who were not accessing these HIV outreach program sites. Similarly, the young people, who did not attend the venues used for recruitment, could not be included. Other studies from different geographical and socio-cultural settings would help in obtaining diverse views and overcoming the aforementioned limitations.

## Conclusion

We conclude that HIV self-test will have acceptability among truckers and young adults in Indian setting. The participants expressed their enthusiasm to use such a test as the results could be quickly obtained and one could do the test on his or her own. However, it will be necessary to address existing apprehensions around HIV self-test and pair such initiatives with interactive strategies to mitigate misconceptions about HIV test and treatment. Community voices captured through the current qualitative investigation underline the necessity of such programmatic initiatives.

## Supplementary Information

**Additional file 1.** Guides for In-depth Interview and Group Discussion.

## Data Availability

Due to the sensitive nature of the questions asked in this study, the survey respondents were assured that the raw data will remain confidential and will not be shared. The data will not be available on public domain. However, if a valid request is made to the corresponding author, the data will be made available through official email communication.

## Abbreviations

CSO: Civil Society Organization
FSW: Female Sex workers
GD: Group Discussion
HIV: Human Immunodeficiency Virus
HIVST: HIV self-test
HIVOST: HIV oral self-test
IEC: Institutional Ethics Committee
ICMR-NARI: Indian Council of Medical Research-National AIDS Research Institute
IDI: In-depth-interviews
IT: Information Technology
MARP: Most at risk population
MSM: men having sex with men
NACP: National AIDS Control Program
PCMC: Pimpri Chinchwad Municipal Corporation
PLHIV: People Living with Human Immuno-deficiency
SHG: Self Help Group
STI: Sexually Transmitted Infections
TI: Targeted Intervention
TR: Truckers
UNAIDS: Joint United Nations Program on HIV/AIDS
WHO: World Health Organization
YB: Young Boys
YG: Young Girls
