# Supplementary material for: Acceptability of HIV oral self-test among truck drivers and youths: a qualitative investigation from Pune, Maharashtra
Source: BMC Public Health. 2021 Oct 24;21:1931. doi: 10.1186/s12889-021-11963-7 (PMC8543938; doi:10.1186/s12889-021-11963-7)
Supplement: Supplementary file 1 — Additional file 1. Guides for In-depth Interview and Group Discussion. [file 12889_2021_11963_MOESM1_ESM.docx]

ADDITIONAL FILE

SUPPLEMENTARY FILE 1 :

Guides for In-depth Interview and Group Discussion

**Interview Guides**

**In-Depth Interview Guide / Group discussion guide**

**Acceptability of indigenous oral HIV self-test : a qualitative investigation**

**Site ___________ Gatekeeper organization________________ Location ________**

**Date DD MM YYYY**

**Start Time**

**End Time**

“Can I seek your permission to switch on the tape-recorder for recording our conversations so that I don’t miss out on anything and play it later to listen to our conversations and learn from them.

(ENCIRCLE BELOW THE RESPONSE FOR RECORDING AS APPROPRIATE)

Yes

No

**Start Recorder if permitted and Read Introduction**:

My name is _________________ (Name of the moderator) and his / her name is __________________ (Name of the note-taker). We will conduct your interview together and keep notes in addition to recording your responses.

Before we begin we would appreciate it, if you could please share something about yourself

- How old are you? …… (Age in completed years)
- Have you ever attended school? --------------
- Which class did you pass in school ? -----------------
- What do you do for a living ?_______
- How much money do you make in a month (in Rupees)?___________
- How long have you been staying in the location where you currently reside?_______

We are interested in knowing your views about issues that will be asked. Please note that there is no wrong or right response. If there are any apprehensions that you would like to talk about you may bring them up even if we haven’t asked them. If there is any question that you would like to ask I would take a note of them and answer them after this interview. If we are unable to answer any of the questions we would refer them to an appropriate person.

As mentioned earlier, the main goal of this discussion is to understand acceptability of oral HIV self-test. We want to remind you that what we discuss here will be kept confidential, and that we will not share your personal information or responses with anyone outside of the study.

(ENCIRCLE BELOW THE RESPONSE FOR RECORDING AS APPROPRIATE)

1. YES
2. NO

If he say “NO” may I know your reasons for declining______________________

Thank you for your response

| **IDI /GD Guide (groups- bridge population, young adults)** |
| --- |
| **1. How comfortable were you in accepting my invitation for participation in this study ?**  *Probes*   - Why do you feel it was important for you to participate? - What benefits do you see of participating? |
| **2. Where do you currently go to seek health care services ?**  *Probe*   - Which facility do you go to? Why? - Please elaborate the reasons for preferring this facility / these facilities - Did you ever take an HIV test before? - If ‘yes’, do you think that had an HIV self-test have been available at the time when you took the HIV test for the first time it would have been useful -please elaborate - If ‘no’ why did you not take an HIV test - How useful do you think an HIV self-test would be to you? |
| 3. **Did you know about HIV self-test earlier than today’s discussion-how did you come to know?**  *Probes*   - Do you think that making an HIV self- test available would be of any help to people? - Please elaborate why do you think so? - What problems do you think people would face to go and take an HIV test from a government facility ? - What is the fear in it? |
| **4. (You said an HIV test would be valuable)-IF SAID IT WOULD NOT BE VALUABLE OR USEFUL DURING DISCUSSION AROUND GUIDE 2 ABOVE-DO NOT ASK THIS- which one would be more acceptable-blood based or oral fluid based ?**  *Probe*  *-Why do you say so?* |
| 5. **If any HIV self -test is made available (show the kit ) will you take the test and do it yourself ? )-IF SAID IT WOULD NOT BE VALUABLE OR USEFUL-DO NOT ASK THIS**  *Probes*   - Please help me understand your concerns - What is the fear? - What would you require for taking such a test? |
| **6. Would you like someone to show how to use the HIV self-test, which I am talking about ?**  *Probes*   - Why do you ask for such assistance ? - What type of assistance would you like - picture, mobile clipping, and individual showing you how to do it? - How could this method be improved ? |
| **7. Where do you think you or your friends would like to take the test ? IF SAID IT WOULD NOT BE VALUABLE OR USEFUL USEFUL DURING DISCUSSION AROUND GUIDE 2 ABOVE -DO NOT ASK THIS**  *Probe*   - Why do you think such a place would be preferred? |
| **8. If you think this test is made available in the market, where would you like them to be available?**  *Probe*   - Please tell me the reasons- why did you say what you said? |
| **9. What are your concerns regarding this test ?**  *Probe*   - Do you think there would be any difficulty in reading the result - Do you think other support services would be necessary (please elaborate) - Do you foresee any individual risk (please elaborate) ? - Do you foresee any social harm (please elaborate) ? - Do you think, some people could use this test in a wicked way ? |
| **10. If this test is made available, do you think there should be a cost attached to it ?**  *Probe*   - What do you think should be an appropriate cost per test kit? |
